# Midwifery

**Published:** 1837-01

**Authors:** 


### MIDWIFERY.

### *A Case of Spontaneous Version.* By Dr. Fr. Niess, of Vöhl.

A healthy stout-made woman, æt. 21, who had had an easy and favorable labour eighteen months previously, enjoyed good health. On the 9th of October, a period which corresponded with her own computation, she first felt slight pains, during which the waters drained very slowly, after the lapse of several hours. A midwife was called in, but the patient would not permit her to make any examination on that day; observing, that the child would soon be born. During the night, when the pains had become stronger, the midwife insisted on making an examination, and, having discovered an arm-presentation, advised her to send for me: this, however, was not done until the following day at noon. When I arrived, about three o'clock in the afternoon, I found the left arm presenting, with the hand protruded from the vulva: the arm was greatly swollen and livid, the palm turned towards the right thigh of the mother. The os uteri had contracted on the arm, so that I could only pass a single finger round it. The womb was closely applied to the body of the child, and I could ascertain, without difficulty, that the head lay over the horizontal ramus of the pubis, towards the right side of the mother, and the feet towards the left side.

According to the midwife's account, the pains had been for several hours violent, frequent, and extremely severe; still the arm made no progress. The general state of the patient was favorable: she merely complained of considerable thirst, and expressed great anxiety about her condition. As I could not think of turning under such circumstances, I proceeded to take proper steps to diminish and regulate the pains. Having premised venesection, as the patient was very robust and plethoric, I gave repeated doses of aqua lauro-cerasi, ordered friction to the abdomen, with a liniment composed of olive-oil and tincture of opium, and tepid oleaginous injections to be frequently thrown up the vagina. Under the continued use of these means, the pains appeared, after the lapse of two hours, to diminish in intensity and severity, and the os uteri had become more yielding; and, as the woman became every moment more impatient and more importunate for assistance, I decided on attempting to turn the child. But, on this and subsequent trials, I found it impossible to pass my hand into the uterus; the irritation produced by attempting to introduce it renewed the pains, and the os uteri was so powerfully contracted on the arm of the child, that a forcible introduction of the hand would have been attended with great risk of rupturing the uterus.

Under these circumstances, and when I found the uterus began to grow more sensible to the touch, I determined to have the advice of another physician, and went to a neighbouring house for the purpose of sending a messenger to him. On my return, somewhat less than an hour afterwards, I was surprised to find the state of things greatly altered. The swollen livid arm, along with the shoulder, had been pushed forwards considerably, and the back had entered the pelvis. With strong pains, accompanied by convulsive tremors of the limbs, the back continued to descend for a quarter of an hour, while the shoulder remained fixed beneath the pubis; and a few minutes after the buttock was evolved, sweeping out under the back with a circular motiou. The lower extremities came away almost at the same instant; soon afterwards the trunk, arms, and head; then the placenta, the expulsion of which was followed by a moderate discharge of blood, which soon ceased.

The child arrived at its full time, and of very considerable size, exhibited no sign of life: the left shoulder, and the parts in its vicinity, were greatly swollen and ecchymosed. The patient went on favorably afterwards· the external parts of generation were at first very much swelled, but she was able to leave her bed on the sixth day.

*Neue Zeitschrift für Geburtskunde,* Band iii. Heft 3.

---

*On Injections through the Umbilical Vein in Cases of retained Placenta.* By Dr. Albert, of Wiesentheil. *With Remarks, by* Dr. D'Outrepont.

Case i. A baker's wife, aged thirty-six, during the first half of her sixth pregnancy, laboured under a slight discharge of blood from the vagina, which was completely arrested by venesection at the end of the fifth month. Towards the end of the thirty-ninth week of gestation, labour came on, and, after four hours, she was delivered of a healthy male infant. Three hours after birth the uterus again enlarged, and a violent hemorrhage took place, so that the patient was found in a state of syncope. Fearing the removal of the placenta would extinguish the remaining spark of life, Dr. Albert filled a small enema syringe with ice-cold water, and injected it through the umbilical vein, ordering the face and region of the heart to be diligently sprinkled and washed with the same. Seven minutes afterwards, he injected half the foregoing quantity. A cold shivering came on, with colicky pains in the abdomen, which disappeared on the supervention of a strong pain, accompanied by contraction of the uterus. While Dr. Albert was preparing to repeat the injection, a second pain came on, and the placenta was expelled. A quantity of coagula were then discharged, and the uterus contracted fully. The patient went on favorably.

Case ii. A girl, of extremely irritable nervous habit, became pregnant in her fifteenth year. During the first half of gestation, she suffered greatly from a constant train of nervous symptoms; during the second, she enjoyed better health than she ever had previously. Towards the end of the eighth month, labour came on: it was extremely slow and painful, and terminated in the birth of a stillborn child. Immediately after the birth of the child, she was attacked with urgent but fruitless desire to pass water, and pains shooting from the spine to the abdomen; the os uteri became spasmodically closed, and could not be opened by any force short of that which would endanger rupture. Dr. Albert, having tried all kind of internal and external antispasmodic remedies in vain, injected the following solution through the umbilical vein:—Lactis tepidi, Infus. Belladonnæ, (ex ℥ss. parati,) āā ℥v.; Extr. Opii Aquosi, gr. vj.; Extr. Hyoscyami, ℨj. (This was the quantity for each injection.) After the first injection the patient became tranquil, and in about twenty minutes fell into a quiet sleep, from which she awoke three-quarters of an hour afterwards, bathed in perspiration. A second injection was now made, and was followed by a copious and extremely offensive alvine evacuation, and the discharge of a large quantity of straw-coloured urine. The patient was greatly relieved; the abdomen became soft, and the os uteri had opened to such a degree that a whole hand could be introduced. On rubbing the belly with a piece of flannel dipped in liq. ammoniæ caust. with tinct. opii, strong after-pains set in, and the placenta was expelled. The after-stage went on regularly.

On the foregoing cases Dr. D'Outrepont makes the following remarks:

"It was for some time a matter of surprise to me to meet so few published cases of injection through the vessels of the funis: reflection and experience have explained the cause. Cold injections through the vessels of the funis possess the disadvantages attendant on all cold injections into the uterus, producing the risk of sudden paralysis, inflammation, and ultimately degenerations of structure. Besides, the detachment of the placenta by cold injections is uncalled for where other and less dangerous means can be employed. This operation, however, may be necessary in certain states of the os uteri; and in this point of view Dr. Albert's second case is of great importance. One of the most disagreeable situations in obstetric practice is where the spasmodic contraction of the os uteri renders the extraction of the placenta impossible. When the usual means of allaying spasm fail, we are authorised in endeavouring to dilate the os uteri; but this is frequently difficult, and even impossible, and in such cases we have to dread the speedy dissolution of the patient. These are the states in which I have seen several women die, notwithstanding the advice and assistance of the most experienced physicians; and it is in such cases that salutary results are to be expected from injections through the umbilical vessels, as by this means we are enabled to bring the antispasmodic remedies to bear immediately on the uterus. The success of this practice is further borne out by the following case, recently observed.

"Case iii. A woman of hysteric habit had hysterical symptoms during delivery, and gave birth to a stillborn child. After the birth of the child, the os uteri became spasmodically closed. The physician in attendance employed frictions over the abdomen with belladonna ointment, injected belladonna into the vagina and rectum, and gave opium internally, but all without effect. Having made a fruitless attempt to dilate the os uteri, I proposed the injection of an infusion of belladonna through the vessels of the cord. The result answered my expectations: after the second injection the spasm yielded, the os uteri dilated, the uterus contracted regularly, and the placenta was expelled.

"Still this experiment is liable to many difficulties. If the injection be made through the veins, the latter are frequently torn, and the fluid remains in the vagina without entering the uterus; when the veins are filled with blood it cannot be accomplished; and injection through the arteries requires a degree of care and attention which cannot be well observed in urgent cases of hemorrhage."

[We insert the foregoing paper more as affording the occasion for warning against, than for recommending the practice. The detail of Dr. Albert's second case is very unsatisfactory, as it does not appear that there was any particular necessity of putting extraordinary measures into execution. We by no means assent to the assertion that spasmodic closure of the os uteri, immediately after delivery, is a frequent occurrence: on the contrary, our experience makes us consider it as a very rare accident under such circumstances. With regard to the practice of injections by the umbilical veins, so long as only cold water or vinegar and water are the fluids employed, we believe it to be free from objection; but we would most strongly dissuade from venturing upon the use of those powerful agents which have been recommended and used by some of our continental brethren,—such as mineral acids; and to the use of belladonna we also entertain very decided objections, having seen it produce, in two instances, its poisonous effects, when introduced only in small quantity into the vagina.] *Neue Zeitschrift für Geburtskunde,* B. iii. Heft 1.

---

*Tubal Pregnancy, ending in Rupture of the Tube.* By Professor BUSCH.

A LADY, aged twenty-four, had been married for several years. In her first pregnancy she aborted in the third month, and was attacked with considerable inflammation of the right ovary, from which she recovered under appropriate treatment. The second pregnancy, which, judging from the corpus luteum afterwards found, originated in the left ovary, went on regularly, and in due time she was delivered naturally of a child come to its full time. This occurred about a year before her death. About seven or eight weeks before death, she again conceived. Her pregnancy, which was recognized by the ordinary signs, was unaccompanied by any circumstance whatever indicating the existence of extra-uterine fœtation. The usual symptoms appeared in regular order; there was no extraordinary pain, and she even went into company the evening before her death. She was there suddenly seized with pains, vomiting, and syncope: the two latter symptoms continued until death. The examination disclosed a rupture of the right fallopian tube, in which the fœtus had been located, and the effusion of a considerable quantity of blood into the cavity of the abdomen.

This case is interesting in many respects, with reference to the doctrine of extra-uterine, and particularly of tubal pregnancies. The first thing that strikes us is the occurrence of the pregnancy in the right tube, as, in far the greatest number of cases of this description, it has been in the left. But it is particularly curious that the very symptoms which we are taught to look upon as the most constant,—namely, the distress from distention, and the consequent colicky pains, &c. as well as the discharge of dark-coloured blood,—were never observed in this case; and that the distention of the tube never produced any inconvenience up to the very period in which rupture took place. Finally, it is not uninteresting to consider the history of the former pregnancies, with reference to the cause of this tubal pregnancy. Although in most cases of extra-uterine fœtation we cannot discover any thing to account for the anomaly, yet in this instance we find, in the history of the previous conceptions, circumstances which enable us to arrive at the probable cause. The first pregnancy, which terminated in abortion, originated in the right ovary, and this organ was attacked with a considerable degree of inflammation, in which, it is very likely, the tube participated. The second pregnancy, which went on regularly to its termination, had its origin in the left ovary; and this third pregnancy proceeded from the right ovary again. These circumstances give a strong colouring to the presumption that, in consequence of the inflammatory affection, the right fallopian tube was either mechanically narrowed, changed in its texture, or deranged in its powers, so as to impede the passage of the ovum into the uterus, and thus give rise to tubal pregnancy.*

*Neue Zeitschrift für Geburtskunde,* Band iii. Heft 2.

---

*On Putrescence of the Uterus.* By Dr. Albert.

Of this obscure affection of the impregnated uterus, first noticed by Boer, Dr. Albert gives the following cases:

Case i. A soap-boiler's wife, fat, bloated, and of a phlegmatic temperament, who had been delivered safely twice before, and never had any remarkable derangement of health, became pregnant the third time, without suspecting it, owing to the absence of her customary symptoms. Towards the end of the ninth month, she became exceedingly weak; her face shrunk and had a clay-coloured hue; the belly was unusually tense and painful on pressure or during the motions of the child, and a dark-coloured, offensive, excoriating fluid was discharged from the vagina. She had scarcely any fever; the pulse was unusually slow and weak; towards evening, however, it became stronger and more rapid; an oppressive feeling of anxiety prevented her from sleeping at night. She had alternate constipation and diarrhœa, no appetite, and little thirst. This state of things continued for eleven days, during which the patient remained partly in bed and partly up. Labour now began, with violent attacks of syncope and slight spasms; the pains soon became strong, but ceased after the head had descended into the pelvis, so that it was necessary to deliver her with the forceps. Dr. Albert had scarcely retired, when he was again summoned, in consequence of the sudden supervention of severe hemorrhage. He found the belly tympanitic, the uterus again enlarged, and a discharge of dark, offensive coagula from the vagina. The state of the patient was utterly hopeless, and she died two hours afterwards. The placenta had come away shortly after the birth of the child.

Thirty-six hours after death, the body was opened. The peritoneum was partially injected, and contained a few ounces of reddish serum; the intestines, particularly the ascending and transverse colon, were distended with offensive gas, and the rectum had a partial reddish tinge. The uterus was externally healthy, its cavity contained clots swimming in a dark fetid fluid. At the left side of the os uteri, commencing at the orifice and running along the cervix up to the fundus, a portion of the internal membrane of the uterus, from seven lines to an inch and three-quarters in breadth, was of an ash-grey colour, and so much softened, that it could be easily rubbed off with the finger. Round this the sound membrane formed a fringed irregular margin. The subjacent parts, as well as the rest of the uterus, the tubes, and ovaries, were quite healthy.

Case ii. A joiner's wife, of small stature and red hair, was delivered (in her third pregnancy,) of a male infant, which died on the sixth day. On the evening of the seventh, she had shivering followed by perspiration, which was kept up by warm drinks, so as to produce great weakness. Towards morning, she had pain with a sense of oppression in the abdomen: this was removed by the diligent application of warm flannels. The following night, she had severe rigors, violent and agonising pain of the abdomen, and a discharge of ill-coloured, offensive lochia.

*The accuracy of the foregoing remark concerning the presence of the corpus luteum appears very questionable. From the researches of Dr. Montgomery, it appears that, even at five months after delivery the corpus luteum has lost so much of its characters as to be recognised only by a well-practised eye; and we very much doubt the possibility of detecting it where more than double that period had elapsed.—Rev.

She complained of oppression about the chest, raved, and frequently attempted to get out of bed. Next morning, her neck, breast, and shoulders were found covered with a miliary eruption; diarrhœa set in, accompanied by excessive weakness; the belly was swelled, the pulse small and rapid, the breathing laboured, the countenance fixed. A dark slimy substance covered the vagina and os uteri, the patient became convulsed, and died six hours afterwards.

On opening the body, the peritoneum was found greatly injected, beset with miliary tubercles, and containing a great deal of serum; the uterus contracted to half its natural volume, externally healthy, internally covered with a dark-brown filthy substance. When this was washed off, the whole lining membrane of the uterus was found putrefied; the subjacent parenchyma of the organ appeared to be quite healthy. The right ovary was twice its natural size, of a livid colour, and firm feel; the back part of the vagina presented a highly inflamed patch about the size of a dollar.

CASE III. An inn-keeper's wife, of stout make and phlegmatic-sanguine temperament, in her second pregnancy, gave birth to two male infants. After delivery, (and even during gestation,) she felt extremely weak, became dejected and anxious, and had a clay-coloured hue. She took no notice of her friends or children, and gave the latter the breast merely because she found the milk troublesome. She slept much, but unquietly, and awoke with incoherence of speech; which, however, ceased when she was spoken to. On the evening of the third day, she complained of weakness, anxiety, and oppression about the præcordia; she had rigors, and the belly became paiuful, so that it was necessary to unloose her bandages; the secretion of milk diminished, the lochia, which were ill-coloured, and offensive, increased. On the fourth day she had diarrhœa and excessive weakness, and died on the following morning. No inspection of the body was allowed. The vagina and os uteri were covered with a dark filthy substance.

CASE IV. A delicate girl, aged sixteen, exposed to considerable privations, became pregnant, and was attacked in the seventeenth week of gestation (having previously enjoyed good health,) with sudden vomiting, constriction of the chest, violent pain shooting from the spine towards the os uteri, syncope, and a discharge of blood from the womb, followed by abortion. Nine days afterwards, while engaged in her occupation as a washerwoman, she was suddenly attacked with syncope, and had a violent hemorrhage from the uterus, after which she fell into a comatose state, and died next morning. At the fundus uteri, close to the entrance of the right fallopian tube, there was a patch about the size of half-a-crown, which had the appearance of viscid dirty-white mucus, and was easily separated from the sound parts by ablution.

Dr. Albert states that he does not know any symptom on which the diagnosis of this affection may be founded, unless the sudden and unusual sinking of the powers of life be admitted as such. At this period, however, the disease is generally beyond the reach of art; and it would be an unfortunate circumstance if experience did not afford us the means of detecting its existence at an earlier period. Dr. Albert thinks, that, as the disease is generally seated about the orifice and neck of the uterus, it may be detected at an earlier period by manual examination, and the use of the speculum. With respect to treatment, he thinks that, in the commencement, and where symptoms of inflammation are present, antiphlogistics may be of advantage; in the latter stage, and where putrescence has occurred, medicated dossils and pledgets (as successfully used by Boer,) are indicated.

*Neue Zeitschrift fur Geburtskunde*, Band iii. Heft 2.

---

*On the Signs of Pregnancy.* By Dr. OSIANDER, of Göttingen.

IT appears that, in Germany, it has been held, on the authority of Stein, who wrote in 1770, that the most certain sign of pregnancy is, that, when it takes place, the os uteri, which was before a transverse fissure, assumes a circular form. This doctrine quite superseded that of Hippocrates, ("quæcunque uterum gestant, his osculum uterorum clausum est,") which, till that time, had been taught in the schools, and which Haller had alone disputed. This assertion of Stein has been propagated as truth by all subsequent German writers on Midwifery. The works of Siebold, Carus, and Hohl, all contain it. In France, on the other hand, Velpeau is the only writer who gives it even partial countenance. The English authorities have never made mention of this fact; and Dr. Osiander has discovered that it is quite imaginary. In its place, however, he has found another sign, to which he is inclined to attach great importance: this he calls the *vaginal pulse.* In pregnancy, he says, the *arteria uterina,* and its branch, the *arteria vaginalis,* must be necessarily increased in size, and their systole and diastole in some degree affected by the process going on in the parts which they supply. At that time he has felt the arteria vaginalis at the posterior border of the columna rugarum anterior, and has found its pulsation to be stronger and harder, and its caliber greater than usual. During imminent abortion and other morbid conditions, he has observed the vaginal pulse to be quicker than the radial.

*Hannoverische Annalen,* B. i. H. 2. 1836.

---

*Successful Cæsarean Operation from an accidental Wound.* By N. E. Pigné. *(Thesis.)*

A woman, æt. 38, in the eighth month of her sixth pregnancy, was gored by a bull, whose horn effected a transverse wound, twenty-seven inches long, running from one anterior and superior iliac spine to the other. The surgeon found his patient cold and insensible, circulation imperceptible, the small intestines lying between the thighs, and covered by a quantity of coagulable blood. They were cleaned; and then was seen the rent in the uterus, from which a male child was spontaneously expelled, and lived for a fortnight. The funis had been ruptured by the fall of the child, and the placenta was extracted through the wound by the assistance of four fingers introduced into the cavity. The intestines were replaced, the edges of the wound in the abdomen brought together, and kept in contact by the interrupted suture. This was covered with charpie spread over with cream, and bandages were placed round the body. The patient did not become conscious until two days after. On the fifth day the lochia appeared. On the twelfth day an eschar, which had formed on the abdominal parietes, separated, and exposed the intestines for an extent of four inches. Nevertheless, by guarding this from the air and using tonics, it cicatrized. In a month she rose from her bed, and has lived twenty years since without any other inconvenience than a small hernia of the left side.

*Archives générales de Médecine, Juillet,* 1836.

